# Report of the Diseases of Edinburgh for November, 1809

**Published:** 1810-01

**Authors:** John Roberton


### *Report of the Diseases of Edinburgh for November*, 1809.

By John Roberton, M. D.

The weather, about the beginning of the month, was soft, and the air moist and disagreeable. Cold unpleasant fogs too prevailed, especially after sun-set, rendering the evenings very unhealthy, particularly to those subject to chronic diseases. This state of the weather was succeeded by smart frost, which continued a week or two, accompanied by cold sharp winds. The surrounding mountains were all covered with snow, which is almost always the case immediately previous to this state of weather at Edinburgh. This, however, in the present instance, has not been the case, as hitherto we have had no snow this season. About the last third of the month, we had frequent heavy falls of rain, accompanied by gales of wind. The mornings again became frosty, and the days soft, but, upon the whole, pleasant till the termination of the month.

The barometer, which stood high about the end of last month, sunk considerably during the thick and foggy days, and even during the frost it was rather low; indeed, throughout the whole month it was low.

The thermometer, except during the frost, when it was very low, stood in general about 40 of Fahrenheit's scale.

The prevailing diseases have been various chronic affections, and, in addition to the acute complaints, such as catarrh, rheumatism, &c. which formerly prevailed, there has, as is very common about this season, been added different inflammatory affections. Fevers and small-pox, I may also remark, have been very malignant and very destructive, especially fevers.

The chronic diseases, which most generally prevailed, were rheumatism, catarrh, and asthma. The too generally unsuccessful treatment adopted in these diseases I had formerly an opportunity of detailing, and it is therefore now unnecessary to repeat it. The acute diseases are, in some measure, similar to those of last month, such as catarrh; the rheumatism too has been frequently met with in practice. The diarrhœa, however, of last month seems to have become less common, nor has it been so severe. Ophthalmia also has not been so frequently met with as might have been expected; indeed, it has almost disappeared. The most commonly to be met with inflammatory complaints are pneumonia, enteritis, and, among chilrden, that inflammatory affection of the brain which generally, unless actively treated, terminates in effusion of water into the ventricles. The cases of pneumonia have not been very general, but they have appeared in some instances with great violence; although they have not in general terminated fatally, they have only been prevented from doing so by the most active treatment. The best proof of this is, that the patients who, from neglect either of themselves or their medical attendants, were not subjected to the active means alluded to, either died or escaped with the greatest difficulty. I have had occasion to order repeated venesection in a very few days, and that that, at each time, the patient should be allowed to bleed till he became faint. The application too of large blisters over the whole forepart of the thorax, with the frequent administration of brisk saline cathartics, were found absolutely necessary to prevent fatal consequences.

The only application which seemed decidedly advantageous in the cases of enteritis, was the frequent application of large blisters over the whole abdomen. Bleeding did not seem nearly so advantageous. I confess, the application of such large blisters as I have alluded to, are very inconvenient and disagreeable; but their speedy efficacy in the removal of the disease is wonderful, inducing a patient to submit to almost any application, however severe.

The cases of inflammatory action of the brain, I have frequently remarked, are most advantageously treated by repeated blistering all over the head, with the frequent administration of brisk purgatives. In short, there seems to be no other method of overcoming that affection but by these means. In severe cases, the application of leeches to the temples, and even opening the temporal artery, is of very great benefit. It is, however, unnecessary for me again to dwell upon this subject.

I have given, I believe, a new view of this universally prevailing and fatal disease, and a very effectual mode of subduing it.

The small-pox seems rather to have increased in severity since last Report. The disease, indeed, is not very general, but some cases of the worst kind occur in practice. Considering the means we possess, of obviating this disease, it is shameful that it should any where exist.

The fevers which prove most troublesome at present are of a slow kind. The symptoms are not violent, but the course of the complaint seems to be of an indefinite length. Thus it wears out those who are its victims, and in many instances, after nearly a month's continuance, it has proved fatal. Those who recover from it are very much reduced in strength, so much so, indeed, as scarcely to give us room to expect a complete restoration of health for several months.

*Princes Street.*

